# Retraction Note: Neutrosophic statistical test for counts in climatology

**DOI:** 10.1038/s41598-022-15750-1

**Published:** 2022-07-04

**Authors:** Muhammad Aslam

**Affiliations:** Department of Statistics, Faculty of Science, King Abdulaziz University, Jeddah, 21551 Saudi Arabia

Retraction of: *Scientific Reports* https://doi.org/10.1038/s41598-021-97344-x, published online 08 September 2021

The Editors have retracted this Article. Following publication, concerns were raised about the rationale for the approach presented and the underlying reasoning. A post-publication review of the authors' mathematical arguments revealed a lack of clarity in the terms presented and inferences that are not adequately justified. The Editors therefore no longer have confidence in the conclusions presented.

Muhammad Aslam does not agree to this retraction.

